# Fatigue of Dental Implants: Facts and Fallacies

**DOI:** 10.3390/dj4020016

**Published:** 2016-05-24

**Authors:** Keren Shemtov-Yona, Daniel Rittel

**Affiliations:** Faculty of Mechanical Engng, Technion, Haifa 32000, Israel; kerenrst77@gmail.com

**Keywords:** fatigue, dental implants, spectrum loading, fracture, complications

## Abstract

Dental implants experience rare yet problematic mechanical failures such as fracture that are caused, most often, by (time-dependent) metal fatigue. This paper surveys basic evidence about fatigue failure, its identification and the implant’s fatigue performance during service. We first discuss the concept of dental implant fatigue, starting with a review of basic concepts related to this failure mechanism. The identification of fatigue failures using scanning electron microscopy follows, to show that this stage is fairly well defined. We reiterate that fatigue failure is related to the implant design and its surface condition, together with the widely varying service conditions. The latter are shown to vary to an extent that precludes devising average or representative conditions. The statistical nature of the fatigue test results is emphasized throughout the survey to illustrate the complexity in evaluating the fatigue behavior of dental implants from a design perspective. Today’s fatigue testing of dental implants is limited to ISO 14801 standard requirements, which ensures certification but does not provide any insight for design purposes due to its limited requirements. We introduce and discuss the random spectrum loading procedure as an alternative to evaluate the implant’s performance under more realistic conditions. The concept is illustrated by random fatigue testing in 0.9% saline solution.

## 1. Introduction

Dental implants are functional structures whose goal is to remediate edentulism [1]. In that respect, dental implants are a subgroup of the larger family of orthopedic implants. The conceptual criteria for successful dental implants should take into account several requirements which are detailed next.

The first is *biocompatibility* which aims at minimizing foreign body reactions by the patient’s immune system. This requirement is of a limiting nature as to the choice of materials that can be considered for the manufacturing of implants in general, and dental implants in particular. As of today, considering metallic materials at that stage, those materials include a few stainless steel grades, magnesium (and some alloys), commercially pure titanium (CP) and its alloys, among which the most widely used is Ti6Al4V [2]. Dental implants are essentially made of CP titanium and Ti6Al4V alloy. A related consideration for material selection is its stiffness. Titanium and its alloys possess a relatively low stiffness (Young’s modulus) when compared with steels, a fact that brings them closer to the stiffness of the bones in which they are implanted. Matching the bone and implant stiffness as much as possible reduces stress gradients, thereby limiting the load transferred to the bone support. In other words, the reduction of the mechanical heterogeneity will make the metallic implant a more “nature-like” addition that does not differ much from its support.

*Osseointegration* is the next important factor, originally defined by Branemark (1952) as a “*direct structural and functional connection between the living bone and the surface of a load bearing implant.*” The definition of osseointegration has evolved since then, and it is now agreed that an implant can be regarded as osseointegrated when there is no progressive relative movement between the implant and the bone with which it is in direct contact. In practice, this means that osseointegration implies an anchorage mechanism whereby artificial components can be reliably and predictably incorporated into living bone, and that this anchorage can persist under all normal loading conditions [3]. Consequently, osseointegration is the main process of interest in implantology, and hence the most widely investigated as of today.

The next factor is that of *clinical manageability*, to ease the surgical procedure as a result of a clever macro-design process. The first goal here should be to ensure primary implant stability as a prerequisite for successful osseointegration. For implants to provide a satisfactory clinical solution, their dimensions (diameter and length) must be made available in various combinations.

The surface condition of the implant is another prime factor for its successful integration [4,5]. Surface roughening of the implants has long established itself as a golden standard, so that several kinds of surface roughening treatments are routinely used nowadays, such as laser processing, special coatings (some of which can be bio-functionalized), and finally grit blasting and acid etching [4]. The latter is by far the most popular treatment today in terms of ease of application. Small ceramic particles (oxides such as titania, zirconia or alumina) are fired at high pressure against the implant’s blank, which they impact and subsequently roughen. The next step consists of proprietary etching to dissolve and remove the particles as much as can be done on the one hand, while adding to the surface roughness on the other hand. Next, the composition of the implant’s surface is examined using spectroscopic techniques to assess its cleanliness in terms of residual contaminants that would negatively affect the osseointegration process. 

Once optimal osseointegration has been achieved, the long-term survival of the implant is of prime concern at two levels, namely biological and mechanical ones.

Biological implant failures are caused by a progressive loss of bone support around the implant because of infection or inflammation termed peri-implantitis [6,7,8]. Approximately 50% of implant losses are defined as late losses, which occur due to loss of bone support [7].

Mechanical complications vary in severity from screw loosening to implant (and its parts) fracture, the rarest of all, which all harm the rehabilitation’s stability and longevity. Implant fracture is the most severe mechanical complication, and therefore the main issue discussed in this paper, with the objective to understand and improve the long-term mechanical reliability of dental implants.

The main mechanical considerations concern the loads as part of the applied constraints experienced by the implant during its service. Some of those loads are related to mastication, on top of which other types of loads should be added, such as those arising from parafunctional habits such as bruxism. Various values of mastication and/or bite loads can be found in the literature [1,9].

Judging from the literature, it appears that there is a very large variability in the reported values of the loads experienced by the implant, to an extent that one can neither define a *characteristic* range of loads, nor coin an *average representative value*. Those loads can range anywhere from 100 to 2400 N [1,9]. This important point will be discussed later in the context of mechanical testing of dental implants *versus* the notion of a “representative load”. 

Likewise, the intraoral environment is widely variable with time and location with respect to the implant. The typical intraoral fluid medium contains different electrolyte concentrations and pH, enzymes, proteins and cells. When the implant’s threads get exposed to the oral environment due to bone loss (perimplantitis), the implant’s body might get exposed to saliva, where food contents (solids and fluids) and temperature are constantly varying [10]. Therefore, here the notion of a “representative environment” is questionable as well, to which one should add that a definite synergy between the intraoral environment and the applied loads can possibly cause a reduction in the implant’s life, as discussed briefly in the sequel. 

The constantly varying nature of the environment and that of the applied loads suggest that the “optimal” design of a dental implant is a serious challenge in view of the so many unknown parameters. 

Let us now review some basic mechanical considerations related to the implant’s fracture as the ultimate limitation to its functionality.

From a structural point of view, fracture can be roughly divided into two types. The first, *immediate* fracture, is always the result of the application of loads that caused the local stresses to exceed the yield (plastic flow) or fracture strength of the component’s material. For this reason, it is referred to as *monotonic* overload, which happens immediately upon application of the load. The most frequent causes for such failures are improper material selection, overlooked stress concentrations, poor processing/machining, and finally excessive use. Monotonic overload failures have not been reported for dental implants to the best of the authors’ knowledge, and will not be further discussed. 

The second kind of fracture mechanism is that of the *time-dependent* failure. As its name suggests, time is the prime factor to be considered in this type of failure. In other words, some kind of damage accumulates over time (and usage), until it reaches a critical level (value) that causes catastrophic failure. Among the various mechanisms that belong to the time-dependent category, one should cite the prominent ones, namely *corrosion* (and its variants) and *fatigue*, as well as their combination [11,12].

Note that each mechanism is rather well defined, and its operation can be ascertained in a process called “failure analysis”, whose main objective is to recommend procedures to avoid additional failures of the same kind [13,14,15].

From here on, we will concentrate on fatigue, as the main fracture mechanism of dental implants. According to the *Encyclopedia Britannica* [16], fatigue is defined as a “*weakened condition induced in metal parts of machines, vehicles, or structures by repeated stresses or loadings, ultimately resulting in fracture under a stress much weaker than that necessary to cause fracture in a single application.”* The word “repeated” is particularly important, because repeated does not mean “cyclic”, as discussed later, but simply means “sustained”. The interested reader will find a wealth of information on fatigue in seminal treatises such as Suresh’s book [11], or in Schijve’s review [17], or simply by browsing through the thousands of articles that have been published over the years in the *International Journal of Fatigue*. 

## 2. Fatigue of Dental Implants

Until recently, fatigue has often been incriminated as the main failure mechanism of dental implants on several occasions [13,14]. However, the results were often weakly supported by the reported physical evidence, as opposed to a recent series of paper by Shemtov-Yona and Rittel [15] who performed a thorough characterization of the fatigue failure of dental implants. Those works reported systematic comparisons between field failures and lab-induced ones that established, beyond any doubt, the operation of a fatigue mechanism in dental implants.

**Figure 1:**
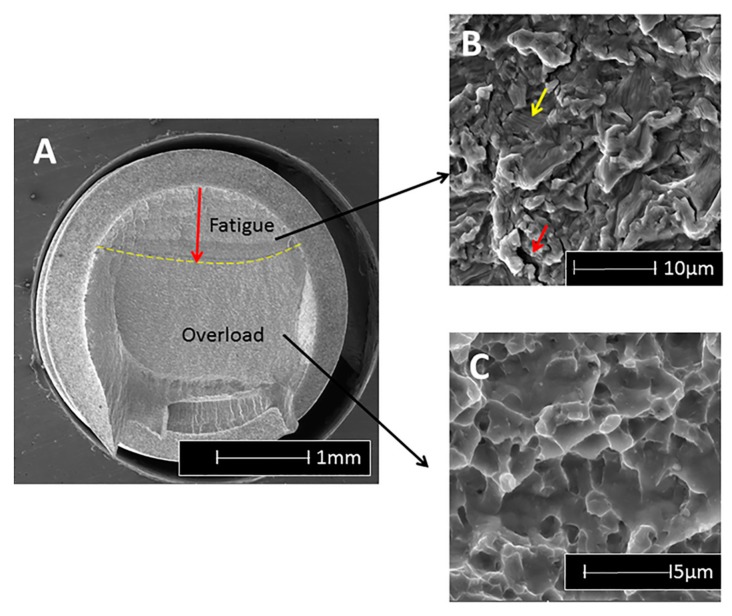
Typical features observable on a fracture surface of a fatigued Ti6Al4V implant, using the scanning electron microscope. (**A**) Overall fracture surface in which two regions are discernable, delineated by the dashed line. The stable fatigue crack, followed by the catastrophic overload fracture; (**B**) In the fatigue region, one can notice faint striations (fine parallel lines) and secondary cracking (arrowed); (**C**) Ductile overload dimples.

As an example, when the fracture surface of titanium and alloy implants is examined using a scanning electron microscope (SEM), the first noticeable feature is the macroscopically different contrast between the stable fatigue crack growth region followed by the catastrophic overload (Figure 1a). In the fatigue region, a characteristic pattern of fine parallel lines (striations) can be discerned to a variable extent, using relatively high magnifications (Figure 1b) [18], while for CP-Ti, the striations are usually quite apparent; in Ti6Al4V it may prove more difficult to detect this pattern. However, in this material, one can discern arrays of parallel microcracks that are perpendicular to the main fracture plane at lower magnifications. Those secondary cracks (Figure 1b) are a clear indication of fatigue failure in this material. Finally, when the fatigue crack becomes unstable, overload fracture ensues, which is characterized by microvoids (dimples), as shown in Figure 1c.

The fatigue striations can be further assessed in terms of their spacing, to provide indications on the load amplitude exerted on the implant, according to what is known as the Paris law [19]. In other words, the “signature” of the fatigue crack growth is well known and documented, and thus easily recognizable. Therefore, one can conclude that the identification of fatigue as a fracture mechanism is well defined nowadays, and is no longer subject to interpretations or guesswork.

With that, the issue of the origin of fatigue cracks has eluded identification for the simple reason that there are several possible causes for fatigue crack initiation. However, it is crucial to know *why and how* fatigue cracks initiate in dental implants so that this stage can be delayed as much as possible. From many private communications, it appears that the practitioners are often blamed for the quality of their implantation. While this may be sometimes be the case, it should be understood that in such a case, implant fracture would not happen years after the implantation by metal fatigue, so other more evident causes must be looked for.

The first identified cause relates to mechanical design considerations, namely the presence of stress concentrations due, e.g., to the threads in relatively thin metal sections [20,21]. The second, much less straightforward, identified the grit-blasting process, which may result in a significant degradation of the metal surface beyond its mere roughening. This degradation consists of surface flaws induced by the impact of ceramic particles, the latter remaining embedded in the surface of the implant [22].

While the end of (stable) fatigue crack growth in a structure is a synonym of catastrophic fracture, it is important to monitor the stable growth process to gain information on the *structural health* of the component (implant). Here, contrary to what exists in other engineering designs, the identification of potential flaws in dental implants *during their service* is almost non-existent. There are obvious reasons for that, starting with the fact that visual inspection is severely limited, and ending with the fact that more powerful techniques such as X-ray inspection, which all necessitate high energies, do present health hazards. One must also note that the small size of the fatigue cracks (less than one millimeter) in dental implants makes them virtually undetectable using conventional inspection techniques. Consequently, as of today, there are no available non-destructive techniques that would allow for early detection of flaws in dental implants. In that context, one should note a recent report from Shemtov-Yona and Rittel [23], who examined the surfaces of retrieved dental implants. All the 100 examined implants had absolutely no external sign of mechanical damage, and they had been retrieved from the oral cavity due to biological complications. However, 62% of those implants were found to be flawed or cracked to a definite extent, with an undeniable involvement of embedded ceramic particles in the generation of surface cracks. Those cracks and flaws, which grew over time, are the clear result of the metal fatigue process. Beyond the surprisingly high number of flawed implants, it must be realized that this study *is the first to date that can be qualified from an* in vivo *assessment of the structural health of dental implants*, since all the implants were extracted for a reason other than mechanical, and were thus apparently intact. As such, there was no record of the implant and patient history, so no correlations could be made in that respect. It is therefore expected that similar future studies for well-categorized implants will be carried out, in order to provide information of the statistical distribution of the time span until crack development with respect to the implant location and patient-related parameters.

## 3. Fatigue Design-Standard Certification and Life Expectancy

**Figure 2:**
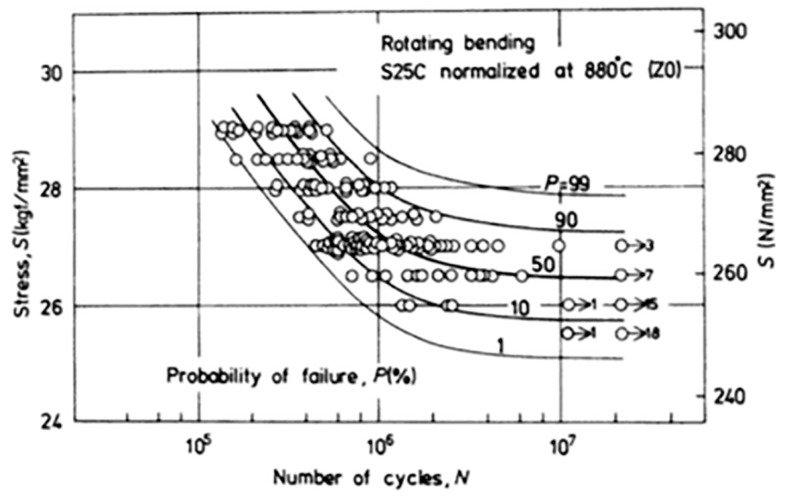
Statistical scatter of S/N curves. Note the various failure probability levels (reprinted with permission from Nishijima, 1981).

Conventional fatigue testing consists of subjecting a specimen to *cyclic* loading at *constant alternating loads*, using tension-tension, bending or other tests [24]. The results are plotted in what is called an S/N or a Wöhler plot, which shows the number of cycles sustained by the specimen as a function of the applied load (or stress), as shown in Figure 2.

It is customary to distinguish three domains in the curve. The first is called the finite life region, in which all the tested specimens fail (fracture) after a finite time. The second, as the load gets lower, is called the transition regime, during which some of the specimens fail while others do not, which are called “runouts”. Here, the test duration is longer, and one customarily sets a limit on the number of cycles, typically five million, to assess the state of the specimen. Finally, as the load is lowered again, one reaches a so-called *fatigue limit*, namely a load (stress) level at which none of the specimens will fail after, e.g., five million cycles. One notes here that the very existence of a fatigue limit has been questioned in a series of papers by Bathias and colleagues [25], when it comes to a very high number of cycles. Still, concerning dental implants, a few million cycles can be deemed to be sufficient and representative. From a purely semantic point of view, one can distinguish between *low cycle fatigue*, for which the specimen sustains up to a few thousand cycles, and *high cycle fatigue*, for which it lasts for several hundred thousand to a million cycles [11]. It is interesting to note that in the low cycle regime, the life duration is distributed as a function of the number of cycles, while in the infinite life regime (fatigue limit), the distribution is about the applied load for a fixed number of cycles [26]. Both high and low cycle fatigue have their own “signature” when examined in the scanning electron microscope (SEM), and can readily be identified [18]. 

However, a key characteristic of fatigue is its inherent statistical scatter, which can sometimes be very large. Considering a typical S/N curve as shown in Figure 2, and noting that it is reported in semi-logarithmic form on the abscissa (number of cycles), one notes that for a fixed level of stress (load), the specimens exhibit a life span that can cover two logarithmic decades! Therefore, the question arises of what is the best way to report fatigue results, or in other words, to which level of confidence does a reported S/N curve correspond? Here comes the first problem when one wants to compare S/N curves originating from, e.g., two different implants. The 90% confidence curve will vastly differ from that plotted using the median points (50%). Incidentally, in reporting levels of confidence, it will be tacitly assumed that Gaussian distributions prevail, a point that may not automatically be satisfied [26,27].

The next problem, relating again to the wide statistical scatter of the results, is that of the representative number of specimens to be tested in order to assure a certain level of confidence. Once a statistical distribution is assumed or ascertained, this number is automatically determined and often reaches 10 or more specimens per stress level.

Another misconception relates to the concept of fatigue (or endurance) limit. While accelerated methods (e.g., staircase, as in [28,29]) have been devised, it should be clearly specified which level of confidence is reported. 

At this stage, it must be clear that a mere “look” at two S/N curves, in order to assess the superiority of one implant type over the second, can be very misleading. The same can be said of a fatigue limit, as long as it has not been proved using a statistical analysis (e.g., analysis of variance), where the compared groups are statistically different for a given level of confidence.

One last but not least point relates to how the designer can make use of data reported as S/N curves to optimize the design of the dental implant. Based on the above, one can think of two approaches. 

The first is based on the fatigue limit concept which “guarantees” an “infinite life” if the load (stress) levels do not exceed it. Such an approach may lead to exceedingly conservative designs aimed at lowering the loads to sometimes unrealistic values. This, of course, does not take into account potential stress concentrations due to the geometry, which may simply go overlooked by the designer.

The second is based on recognizing that in real life, very few components, and certainly not dental implants, are subjected to *constant amplitude cyclic loading*, as discussed earlier. Provided the designer has some idea of the anticipated service load distribution (a very strong assumption), one can use the so-called *cumulative damage approach*, of which Palmgren-Miner’s rule is the best-known example [11]. This rule just states that every load cycle above the endurance limit makes its own contribution to the accumulating (cumulative) damage. Assume that at a given constant cyclic load, a structure can withstand N_f_ cycles to failure. If the same structure is subjected to N_i_ cycles at the same load, with N_i_ ≤ N_f_, the relative contribution of this sequence will be given by N_i_/N_f_. Therefore, the summation of the individual contributions, once it has reached a value of 1, is equivalent to structural failure. Palmgren-Miner’s rule [11] provides a simple way to evaluate the life of a repeatedly loaded component, even if its accuracy has been challenged, as discussed, e.g., by Schijve [17].

As of today, there is no stringent standard for the fatigue testing of dental implants, except for the ISO 14801 standard [30]. In this procedure, the implant is to be tested under what is deemed by the testing laboratory to be the “worst case” conditions experienced by the implant. Those include tilting the implant by a certain angle, usually 30°, with respect to its longitudinal axis, in order to induce bending stresses that are representative of the implant’s inclination in the oral cavity. Moreover, part of the thread may be left outside of the basal grip to mimic bone loss, therefore adding an additional lever that increases the total bending moment. A total of two to three specimens are to be tested for a few constant load levels which go and decrease until a total of three runouts are encountered. Alternatively, one may use accelerated test procedures, such as the kind mentioned earlier, to determine the endurance limit of the implant. Based on the above introduction, it is clear that the ISO 14801 standard is in no way a requirement for the construction and data processing of a full S/N curve. Its goal is essentially to make sure that the fatigue limit is determined, while it may be argued that an absolute number of three runouts out of a given population size will correspond to different rates of survival, according to the tested size. In any case, the ISO standard does not set a mandatory limit on the value of the so-measured endurance limit. The latter is simply reported as part of the requirements to comply with the standards.

It is therefore evident that with such limited information, the designers of dental implants need additional information, whether as a full S/N curve according to the state of the art, or other information as discussed in the sequel. In any case, the few tests carried out as per ISO 14801 are certainly insufficient to attempt and apply any kind of cumulative damage-based estimate.

Therefore, it seems that a paradigm shift is needed which, instead of assuming and/or seeking to establish an “infinite” life for dental implants, would seek to provide more realistic estimates of its service lifetime. The main assumption here, based on a growing body of clinical evidence, is that dental implants and their components ultimately fail by fatigue, once all other biological kinds of failure have been successfully overcome. The question is to determine *when* will they fail, which in turn defines inspection intervals on the one hand, and perhaps preventive extraction or maintenance on the other hand, just as it happens with other aging structures such as aircraft, nuclear plants or others, even if one cannot compare the severity of an aircraft failure with that of a dental implant failure. However, at least both the clinician and the patient should be aware of the finite lifetime of the newly implanted implant in order to match expectations to reality, just as is the case for other common dental procedures. Such information will probably not diminish the rate of mechanical failures, but will certainly put it in the right perspective of anticipated or early abnormal failure.

## 4. Random Spectrum Loading of Dental Implants

The concept of a spectrum loading has long been accepted and recognized mostly but not only in the aeronautical field. The idea is that if one can devise (record) a typical routine sequence of loading, e.g., takeoff, flight and landing for an aircraft, this spectrum can be repeatedly applied to the selected tested component, with the outcome being the expected number of such sequences the component can withstand. Such spectra [31,32] can be used as benchmarks to validate the design of the component. In the field of prosthetic implants, this approach has not been widely applied, even if one can find an interesting example for the hip joint [33]. Here, a typical sequence of walking and stair-climbing was recorded as a representative daily routine and repeatedly applied until failure.

Concerning dental implants and their components, spectrum loading has not been yet considered, to the best of the authors’ knowledge.

In this case, an additional complication arises from the fact that one cannot really devise a representative spectrum, such as for a flight for instance, due to the huge variability in exerted loads, not to mention the intraoral variable environment. Therefore, until further study, a “realistic” mastication spectrum is beyond reach at present.

However, one can consider an alternative solution as follows. First one should keep in mind that the reported range of mastication/biting loads does not exceed 2400 N. So one can safely assume that the implant will experience loads in the 0–2400 N range, at a variable frequency. The sequence of high and lower loads is not determined, so that the simplest approach is to assume they occur *randomly* without any relation to each other. The frequency of mastication/biting is relatively low, so that making use of it would cause the tests to be excessively lengthy. However, it is well known that for metals and alloys, the testing frequency (within a few tens of Hz) does not affect the fatigue response of the material at room temperature, in the absence of self (adiabatic)-heating and subsequent softening effects. In other words, the test frequency can be increased safely to decrease the test duration at room temperature, just as recently reported in the context of ceramic veneers [34]. While the *range* of possible loads, as opposed to constant ones, is now determined, another important upper bound for the limit load is set by the quasi-static strength of the implant itself, which can be markedly lower than 2400 N, for example. 

Here, one must make a decision out of two possibilities, namely applying loads that will eventually reach 2400 N at some stage of the spectrum, irrespective of the implant strength, or, alternatively, assuming that the maximum applied load will always remain inferior to the strength of the implant.

**Figure 3:**
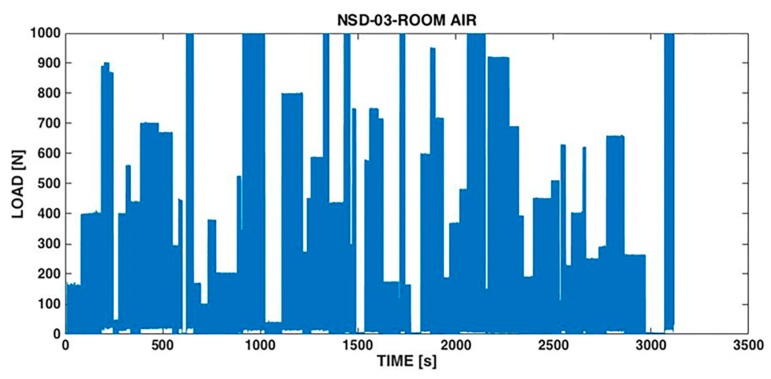
Typical random spectrum of the applied loads. The load varies randomly between 0 and 1000 N at a random frequency in the 0–3 Hz range. Note the presence of pauses during which no load is exerted.

Shemtov-Yona and Rittel [35] developed a random load spectrum testing procedure based on the second possibility, namely applying loads randomly that would not exceed 1000 N, which are slightly inferior to the static strength of their tested implants. A typical spectrum is shown in Figure 3.

In parallel, they performed cyclic loading tests with a peak load of 1000 N. While the cyclic loads failed very rapidly, as a result of low cycle fatigue, the random spectrum-loaded implants lasted about 10 times longer. This result was expected since the spectrum loading comprised many smaller loads which prolong the fatigue life, in the spirit of Palmgren-Miner’s rule. Moreover, it was observed that both the failure locus and the fracture micromechanisms of the implants were identical for cyclically and spectrum-loaded implants alike. This again is expected, based on the concept of the weakest link. Such a link is typically the locus at which the local stress or strain level is maximal and therefore will fail first in the structure. It is expected that irrespective of the character of the applied load (cyclic or spectrum), the weakest link of the implant remains the same. The only difference between the two cases, as mentioned, lies in the rate at which local damage accumulates. 

What comes out of this study is the potential for development and application of a *standardized spectrum*. The latter could be used to test any and all prospective dental implants, with an eventual requirement that the resulting time to failure (and its standard deviation) be bounded to some specific values imposed by the standard. This latter stage would require extensive statistical correlation and follow-up on the mean service life of dental implants in relation with their performance under random spectrum loading. However, at the first stage, one could just have to report the measured values, just as in the current ISO requirement for the fatigue limit estimate. 

However, beyond standardization procedures, the spectrum loading procedure has a definite advantage over cyclic loading. While for the latter *each load level* is characterized by its own standard deviation, for the spectrum loading all the results have *only one standard deviation value*, which is much easier to handle if comparisons must be established between groups of implants. 

Such groups can be of any kind, such as one implant with two different surface treatments whose influence must be assessed or another case in which the thread geometry has been modified to ensure a better implant stability, while there is a definite need to make sure that the change does not affect the fatigue performance of the implant.

While the potential for comparisons using the random load spectrum technique appears to be virtually unlimited, we will now illustrate it in the case of the intra-oral environment and its influence on an implant’s service life.

## 5. Spectrum Loading Performance in Saline Solution

The influence of 0.9% saline solution was evaluated through a series of experiments carried out on monolithic Ti6Al4V implants manufactured by Sigdent, Israel, with a 3.6 mm diameter and 11.5 mm length. A randomly varying load was applied at a randomly varying frequency to 10 implants tested at room temperature and 10 others tested in 0.9% saline solution. The maximum applied load was 1000 N and the maximum frequency was 3 Hz. Both types of tests were carried out using the same load spectrum shown in Figure 3. The static failure load was determined to be 1136.5 ± 57.1 N. As can be noted in Figure 3, the spectrum comprised pauses of variable length during which no load was applied. However, in a liquid environment, the pauses allow for any potentially harmful process (e.g., corrosion) to operate.

The outcome of those tests was that the dry implants all broke after 3423 ± 1515 s, while those tested in the saline solution sustained 2266 ± 695 s. This shows that the life of the implants was significantly reduced by the saline solution, a conclusion confirmed by a statistical comparison between the two groups yielding a *p*-value of 0.026.

Such tests open the way for a thorough evaluation of various intra-oral atmospheres on the mechanical response of dental implants to spectrum loading. Such information could, in principle, be obtained by performing thorough S/N curves and considering specifically the probability of survival of each test at various load levels, as can be found in Shemtov-Yona *et al.* [10]. However, this procedure is lengthy in comparison to directly testing under spectrum loading conditions. 

## 6. Summary

This brief review was aimed at introducing the issue of fatigue failure of dental implants to the clinical community, with emphasis on the state of the art, current limitations and open issues. While the subject of fatigue has long been known and investigated in the structural engineering community, it apparently has not yet found its way to implant manufacturers and to clinicians to its full extent.

The first step was to characterize the environment and the service conditions of dental implants, outlining their diversity and, therefore, difficulty in devising average representative values.

After having defined fatigue fracture and its characteristic features, the current standard requirements were discussed and it was shown that they do not require a full fatigue characterization of the implant, but rather a few selected points and assessment of a fatigue limit, the latter being determined in a rather flexible way. 

Given the limited applicability of the fatigue limit concept to design purposes, another approach was proposed which acknowledges the fact that implants do ultimately break, just like any other structure. It was therefore proposed to subject the implants to random load spectrum loading, in the form of a benchmark test. In other words, if a benchmark test can be devised and accepted as a standard tool, all implants could potentially be evaluated, and eventually ranked, in terms of their performance under such conditions. The question of representativeness of such a spectrum should be further investigated, but at a first stage, time-to-fracture values could indeed be reported the same way a value for the fatigue limit is reported nowadays.

Another great advantage of spectrum loading is that it readily allows for comparisons between different groups of implants. By different groups it is simply meant that the implants differ, e.g., in their design, materials, dimensions, service treatments or test atmosphere, as illustrated here.

## 7. Conclusions

- The vast majority of dental implants fail by a fatigue mechanism.
- Fatigue is easily identifiable as a mechanism, but its causes can be elusive.
- Fatigue design is an engineering specialty in itself, which can benefit dental implants as well.
- ISO 14801 standard requirements are for certification requirements only. They do not provide much relevant information for design purposes.
- Random spectrum loading, as applied in aeronautics and earthquake engineering, should be considered for the characterization and certification of dental implants.
